# Supplementary material for: Utilization of digital tools to enhance COVID-19 and tuberculosis testing and linkage to care: A cross-sectional evaluation study among Bodaboda motorbike riders in the Nairobi Metropolis, Kenya
Source: PLoS One. 2023 Sep 8;18(9):e0290575. doi: 10.1371/journal.pone.0290575 (PMC10490987; doi:10.1371/journal.pone.0290575)
Supplement: S1 Checklist — (DOCX) [file pone.0290575.s001.docx]

STROBE Statement—checklist of items that should be included in reports of observational studies

|  | Item No. | Recommendation | Page  No. | Relevant text from manuscript |
| --- | --- | --- | --- | --- |
| **Title and abstract** | 1 | (*a*) Indicate the study’s design with a commonly used term in the title or the abstract | 6 | This was an interventional cohort study to demonstrate the use of digital platforms in enhancing COVID‑19 and TB testing and linkage to care among Bodaboda riders in the Nairobi Metropolis |
|  |  | (*b*) Provide in the abstract an informative and balanced summary of what was done and what was found | 2 | This study delivered integrated COVID-19 and tuberculosis testing to commercial motorbike (Bodaboda) riders, a population at increased risk of both diseases with limited access to services, in four counties: Nairobi, Kiambu, Machakos and Kajiado.  5663 participants enrolled in the study, 4946 of whom were tested for COVID-19. Ag-RDT positivity rate was 1% but fluctuated widely across counties in line with broader regional trends. Among a subset tested by PCR, positivity was greater in individuals flagged as high risk by the digital tool (8% compared with 4% overall). Of 355 participants tested for tuberculosis, 7 were positive, with the resulting prevalence rate higher than the national average. Over 40% of riders had elevated blood pressure or abnormal sugar levels. The digital tool successfully captured complete end-to-end data for 95% of all participants. This study revealed high rates of undetected disease among Bodaboda riders and demonstrated that integrated diagnosis can be delivered effectively in communities, with the support of digital tools, to maximize access. |
| Introduction | | | |  |
| Background/rationale | 2 | Explain the scientific background and rationale for the investigation being reported | 3-6 | Integration of TB and COVID-19 management has been recommended to mitigate the disruptive impact of the pandemic response on TB services, while increasing efficiencies... Kenya’s Ministry of Health (MoH) has identified Bodaboda riders as a population of interest, due to their increased risk for COVID-19 and the fact that they have not received the same degree of targeted interventions for COVID-19 screening, testing and vaccination as workers in other sectors such as health, education, security, and long-distance truck drivers. It is therefore imperative to create awareness and demand among the Bodaboda riders for COVID-19 and TB testing and include this critical group in healthcare planning and targeted service delivery. |
| Objectives | 3 | State specific objectives, including any prespecified hypotheses | 7 | The primary objective was to evaluate the use of digital tools in conjunction with Ag-RDTs to support decentralized COVID-19 and TB screening, testing, contact tracing and linkage to care of Bodaboda riders in the Nairobi metropolis.  Other secondary objectives were also investigated in the study, including:   1. creating awareness and demand for COVID-19 and TB testing among Bodaboda riders using rider-led mobilization and digital messaging; 2. determining the COVID-19 and TB positivity and co-infection rates among the Bodaboda riders; 3. evaluating the accuracy/reliability of the Ag-RDT compared with RT-PCR. |
| Methods | | | |  |
| Study design | 4 | Present key elements of study design early in the paper | 6 | This was an intervention cohort study to demonstrate the use of digital platforms in enhancing COVID‑19 and TB testing and linkage to care among Bodaboda riders in the Nairobi Metropolis. The Nairobi metropolitan area includes Nairobi County and bordering centres from Kiambu, Machakos and Kajiado Counties. |
| Setting | 5 | Describe the setting, locations, and relevant dates, including periods of recruitment, exposure, follow-up, and data collection | 6, 7, 10 | The study area has a total population of around 9.34 million people, with 125,000 Bodaboda riders registered in Nairobi County and over 100,000 riders across the other three counties.  Mapping of Bodaboda sheds, where riders congregate, and of health facilities was conducted in each county. The location of community-based sampling sites for the study was determined based on proximity to both the sheds and the health facilities.  Participants (Bodaboda riders) were recruited between 21^st^ October 2021 and 1^st^ February 2022 and their samples were collected between the same dates. The authors did not have access to information that could identify individual participants during data collection. |
| Participants | 6 | (*a*) *Cohort study*—Give the eligibility criteria, and the sources and methods of selection of participants. Describe methods of follow-up  *Case-control study*—Give the eligibility criteria, and the sources and methods of case ascertainment and control selection. Give the rationale for the choice of cases and controls  *Cross-sectional study*—Give the eligibility criteria, and the sources and methods of selection of participants | 7 | Eligible participants were registered Bodaboda riders aged ≥18 years, operating within the Nairobi Metropolis, who presented themselves at testing sites. All participants had to provide written consent. Bodaboda riders previously registered in the study and who had been on TB treatment in the last 24 months were excluded from the study. Follow-up of participants was undertaken through county-patient management mechanisms. For participants who tested positive for COVID-19 via PCR, follow-up was undertaken by Sub-County Disease Surveillance Coordinators who managed result communication and linkage to care; participants were followed up for 10–14 days. For TB positive cases, follow-up was carried out through the Sub-County TB Coordinators for purposes of enrolment into care, tracing for individuals in contact with TB cases, and screening and testing of those in contact with the TB case at the household level. |
|  |  | (*b*) *Cohort study*—For matched studies, give matching criteria and number of exposed and unexposed  *Case-control study*—For matched studies, give matching criteria and the number of controls per case |  |  |
| Variables | 7 | Clearly define all outcomes, exposures, predictors, potential confounders, and effect modifiers. Give diagnostic criteria, if applicable | 7-14; Fig 1 | The primary objective was to evaluate the use of digital tools in conjunction with Ag-RDTs to support decentralized COVID-19 and TB screening, testing, contact tracing and linkage to care of Bodaboda riders in the Nairobi metropolis.  Other secondary objectives were also investigated in the study, including: creating awareness and demand for COVID-19 and TB testing among Bodaboda riders using rider-led mobilization and digital messaging;  determining the COVID-19 and TB positivity and co-infection rates among the Bodaboda riders; evaluating the accuracy/reliability of the Ag-RDT compared with RT-PCR. |
| Data sources/ measurement | 8* | For each variable of interest, give sources of data and details of methods of assessment (measurement). Describe comparability of assessment methods if there is more than one group | 7-14 | Sources of data outlined in Fig 1. Sputum samples were collected from those who were identified as presumptive TB cases and samples were sent to the participating health facility laboratories for GeneXpert analysis. All presumptive TB cases were also tested for COVID-19. All the samples were tested using an Ag‑RDT (PANBIO™ COVID-19 Ag RAPID test device), conducted on-site by nurses according to manufacturer’s instructions. Various metrics were computed on the number of participants at each stage of the study, and results from the COVID-19 and TB tests in terms of the number testing positive, negative and inconclusive. Test performance was calculated in terms of sensitivity, specificity, positive predictive value and negative predictive value. Microsoft Excel, R and Stata were used to analyse data. |
| Bias | 9 | Describe any efforts to address potential sources of bias | 11, 12 | Bodaboda sheds were randomized while the riders presented themselves for screening and testing…There was strict adherence to the eligibility criteria…Of participants undergoing COVID-19 testing via the Ag-RDTs, 20% were also randomly selected for confirmatory testing using RT-PCR. |
| Study size | 10 | Explain how the study size was arrived at | 14 | The study area under consideration is made up of 32 sub-counties with approximately 231,500 Bodaboda riders. A sample of Bodaboda riders from all these sub-counties was enrolled in the study. The study aimed to conduct 160 Ag-RDT COVID-19 tests per sub-country, for a total of 5120 tests. The study also aimed to collect 1260 samples for RT-PCR testing. |

Continued on next page

| Quantitative variables | 11 | Explain how quantitative variables were handled in the analyses. If applicable, describe which groupings were chosen and why | 14 | Various metrics were computed on the number of participants at each stage of the study, and results from the COVID-19 and TB tests in terms of the number testing positive, negative and inconclusive. Test performance was calculated in terms of sensitivity, specificity, positive predictive value and negative predictive value. Microsoft Excel, R and Stata were used to analyse data. |
| --- | --- | --- | --- | --- |
| Statistical methods | 12 | (*a*) Describe all statistical methods, including those used to control for confounding | 14 | Data were collected using the digital tool with pre-coded variables. Various metrics were computed on the number of participants at each stage of the study, and results from the COVID-19 and TB tests in terms of the number testing positive, negative and inconclusive. Ag-RDT and RT-PCR test performance was calculated in terms of sensitivity, specificity, positive predictive value and negative predictive value. Microsoft Excel, R and Stata were used to analyse data. Confounding was addressed mainly at the study design stage. |
|  |  | (*b*) Describe any methods used to examine subgroups and interactions | N/A |  |
|  |  | (*c*) Explain how missing data were addressed | 16-17 | These missing test results were excluded from the final data analysis. |
|  |  | (*d*) *Cohort study*—If applicable, explain how loss to follow-up was addressed  *Case-control study*—If applicable, explain how matching of cases and controls was addressed  *Cross-sectional study*—If applicable, describe analytical methods taking account of sampling strategy | N/A |  |
|  |  | (*e*) Describe any sensitivity analyses | N/A |  |
| Results | | | | |
| Participants | 13* | (a) Report numbers of individuals at each stage of study—eg numbers potentially eligible, examined for eligibility, confirmed eligible, included in the study, completing follow-up, and analysed | 15 | A total of 5663 participants visited the sites and underwent a medical check-up and bidirectional screening for COVD-19 and TB. Of those who underwent screening, 4946 (87.3%) met the criteria for testing for COVID-19 only, or both COVID-19 and TB. |
|  |  | (b) Give reasons for non-participation at each stage | 15-16 | Of the 4946 participants eligible for COVID-19 testing, one participant declined to take the test after consenting, citing fears of discomfort from the nasopharyngeal swab procedure. Therefore, 4945 participants were tested for COVID-19 and 95% (4699) had testing results captured in the system. Of those tested, 246 test results were missing due to data entry errors, as data clerks forgot to update results in the app. |
|  |  | (c) Consider use of a flow diagram |  |  |
| Descriptive data | 14* | (a) Give characteristics of study participants (eg demographic, clinical, social) and information on exposures and potential confounders | 15 | A total of 5663 participants visited the sites and underwent a medical check-up and bidirectional screening for COVD-19 and TB. Of those who underwent screening, 4946 (87.3%) met the criteria for testing for COVID-19 only, or both COVID-19 and TB. Table 1 shows the demographics of study participants. The average age of participants was 33.7 years, with 61% of participants aged between 25 and 44 years. Nearly all participants in the study were male (97.1%, n=5498); there were 165 female participants. Most of the participants were from Nairobi County, with the smallest number of participants from Kajiado County. |
|  |  | (b) Indicate number of participants with missing data for each variable of interest | 16 | Of those tested, 246 test results were missing due to data entry errors, as data clerks forgot to update results in the app. In the <5% of cases where the checks did not manage to prevent capture of erroneous data, these fields were identified by study personnel and erroneous data were corrected. |
|  |  | (c) *Cohort study*—Summarise follow-up time (eg, average and total amount) | 7 | For participants who tested positive for COVID-19 via PCR, follow-up was undertaken by Sub-County Disease Surveillance Coordinators who managed result communication and linkage to care within 10–14 days. For TB positive cases, follow-up was carried out through the Sub-County TB Coordinators for purposes of enrolment into care, tracing for individuals in contact with TB cases, and screening and testing of those in contact with the TB case at the household level. (Note: for TB cases, follow-up was expected to continue beyond the duration of the project as the typical treatment period is 6 months; this was managed by the county). |
| Outcome data | 15* | Cohort study—Report numbers of outcome events or summary measures over time | 16 | A high proportion of the 4946 participants who underwent testing for COVID-19 reported symptoms listed on the screening checklist; majority (Fig 3). Among the 4946 participants, 372 also reported symptoms related to both COVID-19 and TB and thus received testing for both. |
|  |  | Case-control study—Report numbers in each exposure category, or summary measures of exposure |  |  |
|  |  | Cross-sectional study—Report numbers of outcome events or summary measures |  |  |
| Main results | 16 | (a) Give unadjusted estimates and, if applicable, confounder-adjusted estimates and their precision (eg, 95% confidence interval). Make clear which confounders were adjusted for and why they were included | 19 | The sensitivity of the Ag-RDT compared with RT-PCR was 27.91% (95% CI: 14.67, 41.45); specificity was 98.39% (95% CI: 97.55, 99.23). The positive predictive value of the Ag-RDT was 46.15% (95% CI: 26.99, 65.31%) and the negative predictive value was 96.51% (95% CI: 95.3, 97.72). |
|  |  | (b) Report category boundaries when continuous variables were categorized | NA |  |
|  |  | (c) If relevant, consider translating estimates of relative risk into absolute risk for a meaningful time period | NA |  |

Continued on next page

| Other analyses | 17 | Report other analyses done—eg analyses of subgroups and interactions, and sensitivity analyses | 17-19 | Subsections: COVID-19 positivity rates; TB positivity rates; Symptoms among those testing positive for COVID-19 or TB |
| --- | --- | --- | --- | --- |
| Discussion | | | | |
| Key results | 18 | Summarise key results with reference to study objectives | 19 | This study demonstrated that a digitally supported community-based testing model can substantially enhance COVID-19 and TB diagnosis and linkage to care among Bodaboda riders, who are at high risk of contracting COVID-19 and TB. The digital tool provided clinical decision support to guide standardized processes for selecting individuals to receive COVID-19 antigen testing, TB diagnosis and/or confirmatory COVID-19 PCR testing...  The tool also provided real-time end-to-end data capture for over 95% of participants, which supported remote monitoring and greater data quality through data validation checks built into the process. This also enhanced linkage to care, as the study was able to submit complete data on patients testing positive for COVID-19 into county health management systems, so the MoH could follow up as needed to enrol patients in home-based care or provide further clinical services if needed. |
| Limitations | 19 | Discuss limitations of the study, taking into account sources of potential bias or imprecision. Discuss both direction and magnitude of any potential bias | 20 | A limitation of the study is that it did not track the outcomes of participants after this step, except for capturing the results of people tested for TB and by PCR for COVID-19, with 100% of these results being captured. |
| Interpretation | 20 | Give a cautious overall interpretation of results considering objectives, limitations, multiplicity of analyses, results from similar studies, and other relevant evidence | 23 | Overall, this study is important in demonstrating the feasibility and value of decentralized testing initiatives, facilitated through digital platforms. |
| Generalisability | 21 | Discuss the generalisability (external validity) of the study results | 23 | Decentralized, community-based testing initiatives, such as the one investigated here, are particularly important for increasing access to care, particularly among high-risk and vulnerable groups who may be less able to access or afford testing otherwise. Our study shows how existing tools used as part of national disease surveillance can be adapted to support successful decentralized testing initiatives. |
| Other information | |  | | |
| Funding | 22 | Give the source of funding and the role of the funders for the present study and, if applicable, for the original study on which the present article is based | Submitted via PLOS ONE submission system as requested by journal | The study was funded by FIND, the global alliance for diagnostics, United Kingdom (FCDO 40105983), Switzerland (81066910), Netherlands (SDD 4000004160), Canada (DFATD 7429348), The Kingdom of Saudi Arabia (FIND – ACT-A DX PARTNERSHIP 20.08.2020), The Rockefeller Foundation (2020 HTH 059), Germany (BMZ Covid-19 Diagnostic and Surveillance Response 27.07.2021), Australia (DFAT 76442), Kuwait (M239/2020), The Government of Portugal and Partners (ANF, BCP, CGF, APIFARMA) and The BlackRock Foundation (Grant Agreement as of April 20, 2022). Medical writing support was funded by FIND, the global alliance for diagnostics, according to Good Publication Practice guidelines.  Beyond the authors listed in this paper, the funders had no further role in study design, data collection and analysis, decision to publish, or preparation of the manuscript. |

*Give information separately for cases and controls in case-control studies and, if applicable, for exposed and unexposed groups in cohort and cross-sectional studies.

**Note:** An Explanation and Elaboration article discusses each checklist item and gives methodological background and published examples of transparent reporting. The STROBE checklist is best used in conjunction with this article (freely available on the Web sites of PLoS Medicine at http://www.plosmedicine.org/, Annals of Internal Medicine at http://www.annals.org/, and Epidemiology at http://www.epidem.com/). Information on the STROBE Initiative is available at www.strobe-statement.org.
