# Supplementary material for: Utilization of digital tools to enhance COVID-19 and tuberculosis testing and linkage to care: A cross-sectional evaluation study among Bodaboda motorbike riders in the Nairobi Metropolis, Kenya
Source: PLoS One. 2023 Sep 8;18(9):e0290575. doi: 10.1371/journal.pone.0290575 (PMC10490987; doi:10.1371/journal.pone.0290575)
Supplement: S1 Table — (DOCX) [file pone.0290575.s002.docx]

**S1 Table. Bidirectional COVID-19 and tuberculosis screening questions**

| COVID-19 | TB |
| --- | --- |
| Cough of any duration | |
| Fever or chills | |
| Chest pain | |
| Shortness of breath or difficulty breathing | Drenching night sweats |
| Fatigue | Unintended weight loss |
| Muscle or body aches | Body mass index (BMI) less than 18.5 |
| Headache | Recent contact with confirmed tuberculosis case |
| New loss of taste or smell |  |
| Sore throat |  |
| Congestion or runny nose |  |
| Nausea or vomiting |  |
| Diarrhoea |  |
| Recent contact with confirmed COVID-19 case |  |
